# The frequency of impairments in everyday activities due to the overuse of the internet, gaming, or smartphone, and its relationship to health-related quality of life in Korea

**DOI:** 10.1186/s12889-020-08922-z

**Published:** 2020-06-18

**Authors:** Yeo-Won Jeong, Young-Ran Han, Sang-Kyu Kim, Han-Seok Jeong

**Affiliations:** 1 Department of Nursing, Dongguk University College of Medicine, 123 Dongdae-ro, Gyeongju-si, Gyeongsangbuk-do 38066 Republic of Korea; 2 Department of Preventive Medicine, Dongguk University College of Medicine, 123 Dongdae-ro, Gyeongju-si, Gyeongsangbuk-do 38066 Republic of Korea; 3 Department of Statistics, Dongguk University, 123 Dongdae-ro, Gyeongju-si, Gyeongsangbuk-do 38066 Republic of Korea

**Keywords:** EQ-5D-3 L, Health-related quality of life, Behavior addictive, Internet, Smartphone

## Abstract

**Background:**

This study aimed to investigate the relationships between the frequency of impairments in daily activities due to the overuse of the Internet, gaming, or smartphones (IGS) and sociodemographic characteristics, social relationships (including family) & activities, psychosocial characteristics, health status, and health-related quality of life (HRQoL) of Korean adults.

**Methods:**

Secondary data from the 2017 Community Health Survey, a large-scale sample survey conducted yearly in South Korea, were analyzed for 190,066 adults over 19 years of age. Three categories were created for impairment groups due to IGS overuse: No Impairment, Mild Impairment, and Moderate-to-Severe groups. And between-group differences were examined using a one-way ANOVA for health status measured with the EQ-5D-3 L and chi-square tests for all categorical dependent variables, which included sociodemographic characteristics, social relationships & activities, and psychosocial factors. The association between frequencies of daily activity impairments due to IGS overuse and the dependent variables were examined using a multivariate logistic regression analysis and a linear regression model.

**Results:**

Approximately 21,345 (11.23%) of the 190,066 participants reported experiencing impairments in daily activities due to IGS overuse at least once in the previous year and the impairments were more severe in males than females. Participants experiencing impairments in daily activities contacted their friends a significantly higher number of times (4 times or more per month) and engaged in leisure activities more frequently (more than once per month) than those without impairments. There was also a significant positive relationship between IGS overuse and stress, depression, suicidal ideation, and suicide attempts. Among participants aged 19–64, impairments in daily activities due to IGS overuse were associated with a lower HRQoL. Conversely, for those aged 65 and over, mild and moderate-to-severe impairments due to IGS overuse were associated with a significantly higher HRQoL.

**Conclusions:**

Increased impairments in daily activities due to IGS overuse may negatively affect mental health. However, among older adults, the frequency of such impairments was positively associated with HRQoL. This finding could be considered to apply interventions with Internet usage or ICT devices for older adults to enhance their quality of life.

## Background

With the development of information and communication technology (ICT) as well as the increased distribution of smartphones [1], the relationship between the Internet, gaming, and smartphones has strengthened. In 2017, the global number of Internet users was estimated to be 3.578 million [2], and virtually all South Korean households (99.5%) have access to the Internet, and such growth in Internet usage should be considered in conjunction with the smartphone distribution rate [2]. For example, in South Korea, the use of smartphones as portable Internet devices increased from 58.3 to 94.1% between 2012 and 2017 [3]. Furthermore, the number of people using smartphones predominantly for leisure activities (e.g., for online gaming, listening to music, watching videos, social networking services [SNS]) instead of communication (e.g. calling and messaging) has increased [4–7]. This phenomenon can be understood from two viewpoints: First, smartphone usage for leisure activities in lieu of desktop computers has been increasing; given their portability, smartphones are increasingly becoming the primary media for accessing the Internet and gaming [3], mainly because it enables people to perform various activities at any place and time [8, 9]. From the user’s viewpoint, such behavior is generalized without distinguishing between smartphone usage for accessing the Internet, gaming, or communication. A previous study suggested that in addition to their original use as communication devices, smartphones are viewed as physical objects for accessing the Internet and all of its content, including gaming [10]. Hence, this study approached the use of the Internet, gaming, and smartphones as one integrated behavior, rather than as distinct behaviors.

Second, the increasing use of smartphones to access the Internet or gaming could also be viewed from the perspective of behavioral addiction. In 2018, the WHO (World Health Organization) released the ICD-11 (11th revision of the International Classification of Diseases), which included the gaming disorder [11]. In 2013, the gambling and Internet gaming disorders were included under non-substance-related disorders and other conditions in the DSM-5 (Diagnostic and Statistical Manual of Mental Disorders 5th edition) [12]. Although researchers seem to have already reached consensus regarding gaming as specific behavioral addictions, such a consensus is lacking regarding the inclusion of Internet or smartphone usage as behavioral addictions [13–15], problem use [10, 16], or another subcategory of specific behavioral addiction (i.e. SNS addictions) [17]. Kwon mentioned that the term “addiction” has been used to refer to a person who is obsessed with a particular activity, thereafter leading to impairments in daily activities similar to those observed among people with substance dependence and/or who have been engaging in repetitive and/or excessive substance use [13]. More specifically, Lin et al. proposed that one of the diagnostic criteria for smartphone addiction was functional impairment that have been present smartphone use having other negative impacts on daily life and resulting in impairments in social relationships, job performance, or school achievement owing to smartphone use [18]. Under the same scope, Lemmense et al. proposed seven criteria for gaming addiction, which also include problems during daily activities [19]. Internet addiction also includes the “loss of significant relationship, job, educational, or career opportunity because of the Internet” as one of the diagnostic criteria [20]. Hence, it seems that impairments in people’s daily life activities due to overuse of the Internet, gaming, and smartphones (hereinafter IGS overuse) is a common component across the existing diagnostic criteria for the addiction disorders related to these behaviors. Therefore, this study focused on impairments in daily activities, especially those that may be related to IGS overuse. Moreover, previous studies have only dealt with the negative effects of IGS overuse in daily life [6, 14, 15], and how often it interferes with daily life activities has yet to be examined; if impairments in daily life activities is a major indicator of IGS addiction, it can be predicted that, the higher the level of the former, the higher its negative effects on health.

IGS overuse has been shown to influence people’s physical and mental health characteristics; it has been associated with gender [21], education [21, 22], married status [21, 22], occupation (e.g. service worker, students, manager etc.) [22, 23], income [21, 22], smoking and drinking [24], smartphone usage time [7, 21], neck pain or discomfort [25], wrist pain [26], back pain [14]; loneliness [6], depression [15], stress [27], the level of social interaction with family and friends or in social activities (e.g. religion) [22, 28, 29], and life satisfaction and quality of life [22, 27, 30–32]. However, most existing research has been conducted with samples of young adults and adolescents [13, 14, 33–35], and there is scant large-scale research with samples of adults from all age groups. Moreover, the relationships between diverse sociodemographic, psychological, social, and quality of life variables have yet to be examined. Therefore, this study tried to explore the diverse factors related to IGS overuse among adults from all age groups.

Another gap in the literature refers to the fact that studies have only assessed the impact of IGS overuse in people’s quality of life regarding life satisfaction and/or subjective well-being factors [27, 31]. Hence, given the aforementioned fact that IGS overuse affects physical, psychological, and social factors, there seems to be a need for research examining the associations between health-related quality of life factors (e.g. physical pain and psychological functioning) and IGS overuse. In that regard, the EQ-5D-3 L, developed by the EuroQol, is a well-validated and comprehensive scale [36] that is used to measure and assess health-related quality of life (HRQoL) factors both in the general population [37] and among those who are physically ill [38, 39]. To the best of the authors’ knowledge, currently, the EQ-5D-3 L has been sparsely used on IGS overuse research, despite the fact that it includes factors (e.g. impairment in daily activities, anxiety/depression, and pain/discomfort) that seem relevant in this context; therefore, research evaluating the use of the EQ-5D-3 L to measure HRQoL in the context of IGS overuse is warranted.

Hence, this study aimed to investigate the relationships between the frequency of impairments in daily activities due to IGS overuse and sociodemographic characteristics, social relationships (including family) & activities, psychosocial characteristics, health status, and HRQoL of Korean adults using the EQ-5D-3 L.

## Methods

### Sample and data collection

To address these study objectives, we conducted a cross-sectional study analyzing secondary data from the 2017 Community Health Survey, which is conducted yearly in Korea [40]; it is a large-scale sampling survey administered by the Korean Centers for Disease Control based on the law for community health, and it aims to evaluate health status among the Korean population aged 19 and older [40]. In 2017, data were collected with the cooperation of 254 public health centers in 17 cities and provinces of South Korea. To sample the target population, a two-stage sampling method was conducted [40, 41]. In the first stage, the primary sample unit was extracted proportionally using the probability sampling method by considering the size of the household and the number of households by housing type (a apartment or a detached-house) in the resident area. In the second stage, among the selected sample, the number of households was identified, and the final sample was extracted through systematic sampling method [40, 41].

**Fig. 1:**
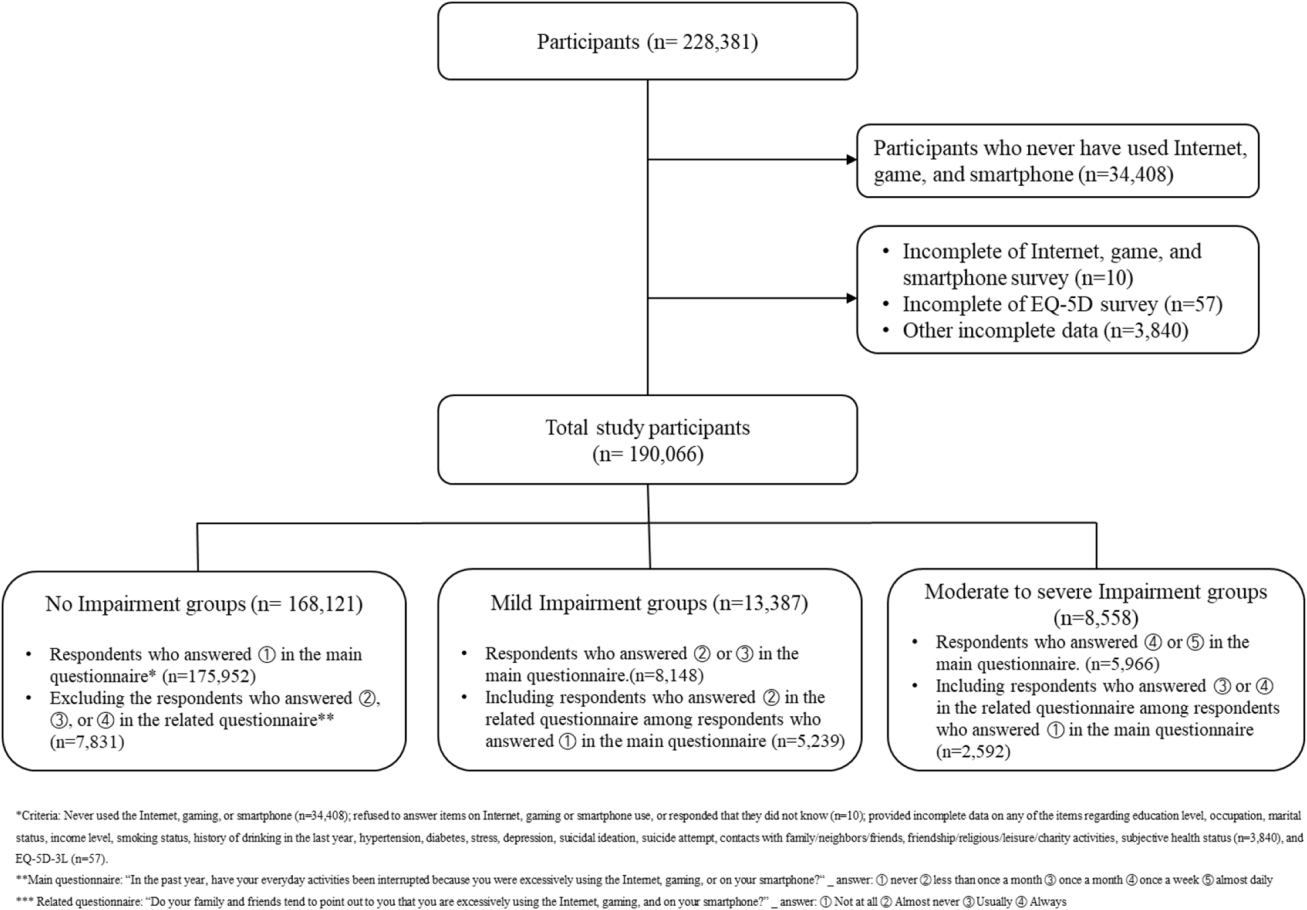
Participants selection process for the present study

After sampling selection, trained field investigators visited each household, explained the purpose of the survey, and then administered it using a computer notebook during one-on-one interviews (computer-assisted personnel interviewing) after obtaining written consent. The questionnaire consists of 201 items across a total of 18 domains, and, since 2013, items relevant to IGS use have been included in the questionnaire every 4 years; that is, data from 2017 represented, at the time of this study, the most recent publicly available data on the topic at hand. In total, 228,381 Koreans participated, but there were exclusions based on the criteria shown in Fig. 1; therefore, the final analytic sample included 190,066 respondents.

Household, individual, and adjusted weights were calculated, and the procedure used to compute weight is presented in Supplementary File 1. Based on the Korean Centers for Disease Control guidelines and previous research [40, 42], a normalized (or standardized) weight was adopted to match sample size. The present study was exempted for approval by the IRB of Dongguk University Gyeongju Campus (IRB No. DGU IRB 20180010–01).

### Variables

In this study, a single question was used to assess the level of impairment owing to IGS overuse: “In the past year, have your daily activities been impaired because you were excessively using the Internet, gaming, or using a smartphone for leisure, not for work or learning purposes?” This question viewed IGS overuse as a behavioral addiction, and approached the construct as a single generalized behavior; regarding such generalization, the use of a single question to assess substance addictions, such as drug and alcohol abuse, has already been validated [43, 44]. For non-substance-use disorders, such as video games or the Internet, single items for self-assessment toward more than one type of addiction behavior have been found to be significantly correlated with an objective assessment of addiction [4, 5, 45]. This item is also one of the questions representing conflict, daily-life, or functional impairment when assessing smartphone, Internet, or gaming addiction [4, 19, 20, 46–48], as well as an item in the 2017 Community Health Survey conducted in South Korea.

Based on previous studies [33, 44, 49], responses to this item should be categorized into one of three groups: No Impairment group (no impairment at all), Mild Impairment group (no more than once a month), and Moderate-to-Severe Impairment group (once a week or almost daily). Moreover, based on previous findings demonstrating that the more severe the IGS addiction, the more frequently friends or family point out the excessive behavior [5, 50], and as an effort to minimize the possible subjective bias of the item, respondents who were initially categorized into the No Impairment group could be moved to the Mild Impairment or Moderate-to-Severe Impairment group depending on their response to Item 7 (“Do your family and friends tend to point out to you that you are excessively using the Internet, gaming, or using your smartphone?” rated on a 4-point scale: *Not at all*, *Almost Never*, *Usually*, and *Always*) in the mental health domain of the survey: Participants who responded *Almost Never* were classified as being in the Mild Impairment group, while participants that responded *Usually* or *Always* were classified as being in the Moderate-to-Severe Impairment group (Fig. 1).

The included variables are shown in Tables 1 and 2, and detailed descriptions of the variables, sociodemographic characteristics, social relationships (including family) & activities, and psychosocial factors are presented in Supplementary File 2.

**Table 1:** Proportion of impairment levels in daily activities due to the overuse of the internet, gaming, or smartphone according to age groups

Age (Years)	Participants who used the Internet, gaming, or smartphones at least once before (*N* = 190,066)			
	No Impairment groups^a^ (*n* = 168,121)	Mild Impairment groups^b^ (*n* = 13,387)	Moderate-to-Severe Impairment groups^c^ (*n* = 8558)	χ2 Post Hoc^2)^
Total	168,121(84.89)^1)^	13,387(9.30)	8558(5.81)	14,299.550^*^a > b > c
19–29	14,951(62.68)	5143(22.65)	3436(14.67)	6635.188^*^a > b > c
30–39	22,779(77.83)	3897(13.85)	2306(8.32)	17,114.821^*^a > b > c
40–49	33,738(88.78)	2623(7.35)	1419(3.87)	33,491.711^*^a > b > c
50–59	38,859(95.07)	1137(2.96)	802(1.97)	39,889.012^*^a > b > c
60–69	30,894(96.81)	446(1.62)	430(1.57)	27,631.206^*^a > b, c
70–79	19,628(98.32)	121(0.79)	143(0.89)	16,275.763^*^a > b, c
80 or older	7272(99.24)	20(0.35)	22(0.41)	5023.811^*^a > b, c

^a:^ No Impairment groups / ^b:^ Mild Impairment groups / ^c:^ Moderate-to-Severe Impairment groups

^1)^ The numbers in parentheses are the row percentages reflecting weight

^2)^ The Bonferroni test was used for Post-hoc analysis

^*^p < .0001

**Table 2:** Sociodemographic factors, socio-family relations, psychosocial factors, health status, and health-related quality of life according to the frequency of impairments in everyday activities due to the overuse of the internet, gaming, or smartphone

Parameters	Participants who used the Internet, gaming, or smartphone at least once before					
	Total (*N* = 190,066)	No Impairment groups (*n* = 168,121)	Mild Impairment groups (*n* = 13,387)	Moderate -to- severe Impairment groups (*n* = 8558)	χ2 or F	Cramer’s V or r^2^
Sociodemographic factors						
Gender					277.029^*^	0.038
Male	87,836(50.27)	76,292(83.03)^a^	6963(10.37)	4581(6.60)		
Female	102,230(49.73)	91,829(86.77)	6424(8.23)	3977(5.00)		
Age					12,181.045^*^	0.178
19–39	52,512(37.54)	37,730(70.28)	9040(18.24)	5742(11.49)		
40–64	78,578(42.28)	89,940(92.45)	4061(4.78)	2480(2.77)		
65 or older	58,976(20.18)	40,451(98.13)	286(0.83)	336(1.04)		
Education					2790.673^*^	0.086
No formal or Elementary school	33,398(9.42)	32,949(98.15)	207(0.86)	242(0.99)		
Middle school	20,344(7.80)	19,633(95.68)	357(2.19)	354(2.12)		
High school	69,425(38.75)	60,000(82.92)	5568(10.15)	3857(6.93)		
College or higher	66,899(44.03)	55,539(81.88)	7255(11.62)	4105(6.50)		
Marital status					8704.693^*^	0.151
Married and living with a spouse	130,531(65.33)	119,672(89.81)	6707(6.35)	4152(3.84)		
Divorced or separated or Widowed	26,079(10.22)	25,213(95.52)	501(2.64)	365(1.84)		
Never been married	33,456(24.45)	23,236(67.29)	6179(19.98)	4041(12.72)		
Average monthly household income					549.227^*^	0.038
Lowest	27,484(8.34)	26,360(92.37)	584(4.16)	540(3.47)		
Low-middle	63,168(28.84)	56,729(86.02)	,735(8.26)	2704(5.72)		
Upper-middle	58,155(34.40)	50,075(83.73)	4986(10.05)	3094(6.22)		
Highest	41,259(28.42)	34,957(82.96)	4082(10.97)	2220(6.08)		
Occupation					4623.842^*^	0.11
Manager, professional or administrator, clerk	43,240(28.97)	36,219(82.51)	4629(11.56)	2392(5.93)		
Service worker, salesperson	26,904(14.65)	23,661(84.91)	1946(9.02)	1297(6.08)		
Technician, mechanic or production worker, machine operator/assembly worker	20,150(11.97)	17,874(87.16)	1418(8.08)	858(4.75)		
Skilled agricultural/forestry/fishery worker, unskilled worker, soldier	35,584(10.93)	33,558(90.99)	1155(5.19)	871(3.81)		
Student	6,371(5.26)	3,591(55.21)	1665(27.25)	1115(17.53)		
Unemployed	57,817(28.23)	53,218(89.52)	2574(5.9)	2025(4.58)		
Location of residence					259.947^*^	0.037
Rural (town, township)	74,466(17.25)	68,474(88.15)	3451(6.82)	2541(5.03)		
Urban (city)	115,600(82.75)	99,647(84.21)	9936(9.82)	6017(5.97)		
Smoking					314.734^*^	0.029
Non-smoker	120,621(62.02)	106,506(84.35)	8780(9.83)	5335(5.83)		
Past smoker	34,094(17.25)	31,186(89.04)	1731(6.58)	1177(4.38)		
Current smoker	35,351(20.74)	30,429(83.06)	2876(10.00)	2046(6.94)		
Alcohol during the last year					535.554^*^	0.101
Yes	136,395(77.90)	117,536(82.97)	11,590(10.53)	7269(6.50)		
No	53,671(22.10)	50,585(91.67)	1,797(4.96)	1,289(3.37)		
Social relationships & activities						
Family contact (by meeting in person or calling)					257.147^*^	0.026
≤ 1 time per month	54,918(32.95)	47,219(82.45)	4645(10.65)	3054(6.90)		
2–4 times per month	53,446(28.71)	47,400(85.72)	3885(9.23)	2161(5.05)		
2–3 times per week	30,682(14.24)	27,682(86.67)	1802(8.08)	1198(5.25)		
≥ 4 times per week	51,020(24.11)	45,820(86.18)	3055(8.27)	2145(5.55)		
Neighbor contact (by meeting in person or calling)					1466.542^*^	0.062
≤ 1 time per month	80,961(56.44)	67,718(81.76)	8077(11.16)	5166(7.08)		
2–4 times per month	28,466(15.14)	24,923(85.15)	2304(9.88)	1239(4.97)		
2–3 times per week	25,071(10.97)	22,824(89.04)	1396(6.92)	851(4.05)		
≥ 4 times per week	55,568(17.45)	52,656(92.17)	1610(4.30)	1302(3.53)		
Friend contact (by meeting in person or calling)					553.135^*^	0.038
≤ 1 time per month	58,449(29.51)	53,176(88.31)	3175(7.03)	2098(4.66)		
2–4 times per month	50,084(28.37)	44,066(85.27)	3871(9.56)	2147(5.17)		
2–3 times per week	34,188(18.32)	29,685(82.98)	2809(10.69)	1694(6.34)		
≥ 4 times per week	47,345(23.80)	41,194(81.68)	3532(10.75)	2619(7.57)		
Religious activity					374.172^*^	0.044
< 1 time per month	139,684(74.18)	122,143(83.60)	10,728(10.11)	6813(6.29)		
≥ 1 time per month	50,382(25.82)	45,978(88.60)	2659(6.99)	1745(4.41)		
Friendship activity					1027.635^*^	0.074
< 1 time per month	83,310(46.56)	71,189(81.01)	7337(11.55)	4784(7.44)		
≥ 1 time per month	106,756(53.44)	96,932(88.27)	6050(7.35)	3774(4.38)		
Leisure activity					65.000^*^	0.018
< 1 time per month	132,286(65.84)	118,294(85.46)	8369(8.77)	5623(5.77)		
≥ 1 time per month	57,780(34.16)	49,827(83.80)	5018(10.33)	2935(5.88)		
Charity activity					117.601^*^	0.025
< 1 time per month	173,194(91.92)	152,803(84.54)	12,461(9.52)	7930(5.95)		
≥ 1 time per month	16,872(8.08)	15,318(88.91)	926(6.86)	628(4.23)		
Psychosocial factor						
Stress					768.859^*^	0.045
No stress	39,319(17.23)	36,687(90.03)	1471(5.68)	1161(4.29)		
Some stress	104,260(56.24)	92,033(85.12)	7810(9.59)	4417(5.29)		
Moderate stress	40,320(22.89)	34,343(81.46)	3544(11.05)	2433(7.49)		
Severe stress	6167(3.64)	5058(78.58)	562(11.04)	547(10.39)		
Depressive mood					170.022^*^	0.030
Yes	11,630(6.32)	9806(79.72)	990(11.26)	834(9.02)		
No	178,436(93.68)	158,315(85.24)	12,397(9.17)	7724(5.59)		
Suicide ideation					58.732^*^	0.018
Yes	13,462(6.43)	11,792(82.50)	891(9.56)	779(7.93)		
No	176,604(93.57)	156,329(85.05)	12,496(9.28)	7779(5.66)		
Suicide attempt					15.510^**^	0.009
Yes	619(0.30)	526(80.79)	39(8.13)	54(11.09)		
No	189,447(99.70)	167,595(84.90)	13,348(9.31)	8504(5.79)		
Health status and HRQoL^b^						
Hypertension					1634.772^*^	0.093
Yes	44,597(18.18)	42,759(94.22)	1030(3.37)	808(2.41)		
No	145,469(81.82)	125,362(82.82)	12,357(10.62)	7750(6.56)		
Diabetes mellitus					703.817^*^	0.061
Yes	18,025(7.33)	17,314(94.91)	398(2.90)	313(2.19)		
No	172,041(92.67)	150,807(84.10)	12,989(9.81)	8245(6.09)		
Subjective awareness of health status					556.087^*^	0.038
Very good	11,897(7.25)	9,999(81.18)	1128(11.37)	770(7.45)		
Good	63,583(36.13)	55,307(83.85)	5398(10.60)	2878(5.55)		
Average	82,260(43.94)	72,458(84.83)	5897(9.21)	3905(5.97)		
Poor	25,977(10.47)	24,202(89.15)	888(5.38)	887(5.47)		
Very poor	6349(2.21)	6155(95.17)	76(1.77)	118(3.06)		
EQ-5D-3 L (M ± SD)^c^						
Total		0.954 ± 0.0003	0.970 ± 0.0006	0.959 ± 0.0010	211.81^*^	0.0022
19–39	52,512(37.54)	0.980 ± 0.0003	0.973 ± 0.0007	0.965 ± 0.0010	212.14^*^	0.0080
40–64	78,578(42.28)	0.962 ± 0.0003	0.962 ± 0.0013	0.948 ± 0.0023	42.13^*^	0.0009
65 or older	58,976(20.18)	0.874 ± 0.0012	0.915 ± 0.0090	0.888 ± 0.0119	11.17^*^	0.0005

^*^p < .0001

^**^p < .0005

^a^ The numbers in parentheses are the row percentages reflecting weight

^b^ Health-related quality of life

^c^*M* mean, *SD* standard deviation

Regarding health status, the included variables considered major physical illnesses that were shown to influence HRQoL (i.e. hypertension and diabetes) [39]. For these variables, if the mentioned diagnosis came from a physician (not self-assessment), respondents were coded as having a health problem. Subjective awareness of health status was assessed through the item, “What do you usually think of your health?” rated on a 5-point scale—*Very good*, *Good*, *Average*, *Poor*, and *Very poor*. Regarding HRQoL, it was measured using the Korean version of the EQ-5D-3 L; it was translated following the guidelines outlined by the EuroQol group [51]. The Korean version of the EQ-5D-3 L has been shown to have high reliability and validity, and has been previously used to evaluate HRQoL in Korean adults [52, 53]. The instrument has five domains: mobility, self-care, daily activities (e.g. work, study, housework, family, or leisure activity), anxiety/depression, and pain/discomfort, and items are rated on a 3-point scale (1 = *no problem*, 2 = *some problem*, 3 = *severe problem*) [51]. The score was calculated using the time trade-off values set by the Korean version of the EQ-5D-3 L [54].

### Analysis

Initially, to show the distribution of all studied variables by the three impairment groups, we examined all descriptive statistics. To examine between-group differences, a one-way ANOVA was conducted on HRQoL and a chi-square test on all categorical variables—including age groups. To measure the differences among the age groups by the three impairment groups, we used the Bonferroni test for post-hoc analysis. Additionally, since Type 1 errors are influenced by sample size, this study calculated the effect size, Cramer’s V and r^2^ to examine the magnitude of the differences [55–57]. The interpretation of the effect size was based on previous studies [55, 57], and Cramer’s V was interpreted by taking into account the degrees of freedom [57].

To determine the association between frequencies of daily activity impairments due to IGS overuse and the dependent variables, we used a multivariate logistic regression analysis and a linear regression model. Adjusted odds ratios were reported with a 95% confidence interval, and statistical significance was determined by *p* < 0.05. All statistical analyses were conducted using SAS software, Version 9.4 Copyright© 2013 (SAS Institute Inc., Cary, NC, USA).

## Results

Approximately 11.5% (*n* = 21,945) of the 190,066 respondents experienced impairments in daily activities at least once in the year prior to the survey, and approximately 38.9% (*n* = 8558) experienced such impairments more than once a week. Slightly over half of adults aged 19–29 were in the No Impairment group, and the proportion in the Mild Impairment group was higher than in the Moderate-to-Severe Impairment group. With increasing age, the proportion of adults in both the Mild and Moderate-to-Severe Impairment groups decreased. For age groups under 60, the proportion of adults was significantly lower in the Moderate-to-Severe Impairment group than in the Mild Impairment group. However, for adults aged 70 and older, this pattern differed slightly: there was a somewhat higher number of individuals in the Moderate-to-Severe Impairment group than in the Mild Impairment group, although these results were not statistically significant (Table 1).

### Differences in the studied variables by the frequency of impairments in daily activities due to IGS overuse

Significant differences among the Mild and Moderate-to-Severe Impairment groups were found for age, marital status (Cramer’s V was medium to large), education level, occupation, history of drinking, contacts with neighbors, friendship activities, stress, and hypertension (Cramer’s V was small to medium). The results are detailed in Table 2.

### Associations between the frequency of impairments in daily activities due to IGS overuse and studied variables

Regarding sociodemographic characteristics in the Mild Impairment group, the frequency of impairments was 1.317-fold (95% CI = 1.278–1.357, *P* < 0.0001) higher for men compared to women; among those who lived in an urban area (AOR = 1.507, 95% CI = 1.442–1.575, *P* < 0.0001), as college or higher education (AOR = 16.257, 95% CI = 13.992–19.036, P < 0.0001) and monthly income increased (AOR = 2.931, 95% CI = 2.711–3.176, *P* < 0.0001 for Highest), the frequency of impairments increased significantly. Particularly, occupation was significantly associated with the frequency of impairments, and students were the group with most severity (AOR = 8.645, 95% CI = 8.035–9.037, *P* < 0.001 in Mild Impairment groups).

Regarding smoking and drinking, the adjusted odds ratio for the Moderate-to-Severe Impairment group was 1.034 for current smokers, and for the Mild Impairment group it was 2.347 among those with a history of drinking in the year prior to the survey.

Regarding social relationships (including family), the odds of contacting families or neighbors 4 times or more per month were significantly lower by 0.743-fold (95% CI = 0.714–0.774, *p* < 0.001) in the Mild Impairment groups compared to the No Impairment groups. On the contrary, contacting friends for 4 times or more per month, including calling or meeting in person, was significantly higher by 1.653-fold (95% CI = 1.585–1.725, *p* < 0.0001) in the Mild Impairment groups and 1.756-fold (95% CI = 1.670–1.847, p < 0.0001) in the Moderate-to-Severe Impairment groups compared to the No Impairment groups (Table 3).

**Table 3:** Adjusted odds (AOR) ratios and 95% confidence (CIs) intervals for the associations of the frequency of impairments in daily activity due to overuse of the internet, gaming, or smartphone with the study covariates using multivariate logistic regression analysis

Parameter	Participants that had used the Internet, gaming, or smartphone at least once before (*N* = 190,066)			
	Mild Impairment groups (*n* = 13,628)		Moderate- to -severe Impairment groups (*n* = 8753)	
	AOR (95% CI)^a^	B (*p*-value)	AOR (95% CI)	B (*p*-value)
Sociodemographic factors				
Gender				
Male	1.317(1.278–1.357)	324.507 (< 0.001)	1.380(1.330–1.433)	285.678 (< 0.001)
Female	1		1	
Age				
19–39	30.665(26.977–35.059)	5124.570 (<.0001)	15.436(13.745–17.410)	4474.538 (<.0001)
40–64	6.115(5.370–7.004)	7.392 (0.0066)	2.827(2.508–3.200)	84.831 (<.0001)
65 or older	1		1	
Education				
No formal or Elementary school	1		1	
Middle school	2.624(2.184–3.165)	198.499 (<.0001)	2.195(1.840–2.626)	101.196 (<.0001)
High school	14.023(12.065–16.428)	1687.220 (<.0001)	8.267(7.178–9.582)	1199.201 (<.0001)
College or higher	16.257(13.992–19.036)	2252.291 (<.0001)	7.848(6.815–9.094)	1070.911 (<.0001)
Marital status				
Married and living with a spouse	1		1	
Divorced or separated or Widowed	0.391(0.358–0.426)	1438.133 (<.0001)	0.451(0.406–0.500)	871.255 (<.0001)
Never been married	4.202(4.073–4.335)	5843.150 (<.0001)	4.423(4.256–4.597)	3987.608 (<.0001)
Average monthly household income				
Lowest	1		1	
Low-middle	2.128(1.966–2.308)	11.728 (<.0001)	1.770(1.622–1.936)	26.005 (<.0001)
Upper-middle	2.661(2.463–2.883)	374.574 (<.0001)	1.978(1.814–2.160)	143.679 (<.0001)
Highest	2.931(2.711–3.176)	650.912 (<.0001)	1.950(1.786–2.132)	112.336 (<.0001)
Occupation				
Manager, professional or administrator, clerk	2.453(2.302–2.616)	171.681 (<.0001)	1.715(1.590–1.852)	9.419 (0.0021)
Service worker, salesperson	1.860(1.733–1.997)	26.883 (<.0001)	1.708(1.572–1.856)	6.836 (0.0089)
Technician, mechanic or production worker, machine operator/assembly worker	1.625(1.508–1.751)	121.444 (<.0001)	1.302(1.190–1.425)	153.007 (<.0001)
Skilled agricultural/forestry/fishery worker, unskilled worker, soldier	1		1	
Student	8.645(8.035–9.307)	4989.934 (<.0001)	7.579(6.960–8.259)	3564.683 (<.0001)
Unemployed	1.155(1.079–1.237)	1152.451 (<.0001)	1.220(1.129–1.321)	414.351 (<.0001)
Location of residence				
Rural (town, township)	1		1	
Urban (city)	1.507(1.442–1.575)	329.357 (<.0001)	1.242(1.180–1.309)	67.792 (<.0001)
Smoking				
Non-smoker	1		1	
Past smoker	0.634(0.606–0.664)	408.154 (<.0001)	0.713(0.674–0.753)	235.488 (<.0001)
Current smoker	1.034(0.996–1.072)	170.912 (<.0001)	1.209(1.157–1.264)	227.175 (<.0001)
Alcohol during the last year				
Yes	2.347(2.224–2.456)	1367.392 (<.0001)	2.130(2.018–2.251)	735.752 (<.0001)
No	1		1	
Social relationships & activities				
Family contact (by meeting in person or calling				
≤ 1 time per month	1		1	
2–4 times per month	0.834(0.803–0.865)	2.201 (0.1379)	0.704(0.671–0.738)	49.249 (<.0001)
2–3 times per week	0.721(0.687–0.757)	52.659 (<.0001)	0.725(0.683–0.769)	17.419 (<.0001)
≥ 4 times per week	0.743(0.714–0.774)	45.191 (<.0001)	0.770(0.733–0.808)	2.670 (0.1022)
Neighbor contact (by meeting in person or calling				
≤ 1 time per month	1		1	
2–4 times per month	0.851(0.816–0.887)	281.895 (<.0001)	0.674(0.637–0.712)	9.269 (0.0023)
2–3 times per week	0.569(0.539–0.601)	28.402 (<.0001)	0.525(0.489–0.563)	43.771 (<.0001)
≥ 4 times per week	0.342(0.324–0.361)	867.876 (<.0001)	0.442(0.416–0.469)	213.733 (<.0001)
Friend contact (by meeting in personor calling)				
≤ 1 time per month	1		1	
2–4 times per month	1.409(1.351–1.469)	0.783 (0.3761)	1.149(1.090–1.211)	62.563 (<.0001)
2–3 times per week	1.618(1.546–1.693)	112.472 (<.0001)	1.447(1.368–1.530)	32.594 (<.0001)
≥ 4 times per week	1.653(1.585–1.725)	176.111 (<.0001)	1.756(1.670–1.847)	366.367 (<.0001)
Religious activity				
< 1 time per month	1		1	
≥ 1 time per month	0.653(0.629–0.678)	502.265 (<.0001)	0.661(0.631–0.692)	307.693 (<.0001)
Friendship activity				
< 1 time per month	1		1	
≥ 1 time per month	0.584(0.566–0.602)	1224.633 (<.0001)	0.540(0.520–0.561)	1026.847 (<.0001)
Leisure activity				
< 1 time per month	1		1	
≥ 1 time per month	1.200(1.164–1.238)	135.328 (<.0001)	1.039(0.999–1.080)	3.690 (0.0547)
Charity activity				
< 1 time per month	1		1	
≥ 1 time per month	0.686(0.644–0.729)	142.164 (<.0001)	0.677(0.626–0.731)	96.597 (<.0001)
Psychosocial factor				
Stress				
No stress	1		1	
Some stress	1.786(1.701–1.876)	9.987 (0.0016)	1.304(1.232–1.382)	194.405 (<.0001)
Moderate stress	2.150(2.038–2.268)	212.038 (<.0001)	1.931(1.816–2.054)	93.078 (<.0001)
Severe stress	2.226(2.043–2.424)	84.269 (<.0001)	2.774(2.533–3.036)	321.368 (<.0001)
Depressive mood				
Yes	1.314(1.240–1.390)	88.804 (<.0001)	1.726(1.619–1.838)	285.274 (<.0001)
No	1		1	
Suicide ideation				
Yes	1.062(1.000–1.127)	3.849 (0.0498)	1.445(1.352–1.543)	118.844 (<.0001)
No	1		1	
Suicide attempt				
Yes	0.920(0.678–1.214)	0.3151 (0.5746)	2.012(1.549–2.571)	29.325 (<.0001)
No	1		1	
Health status and HRQoL^b^				
Hypertension				
Yes	0.279(0.263–0.295)	1851.115 (<.0001)	0.323(0.301–0.346)	1038.598 (<.0001)
No	1		1	
Diabetes mellitus				
Yes	0.262(0.238–0.288)	744.868 (<.0001)	0.318(0.284–0.354)	412.470 (<.0001)
No	1		1	
Subjective awareness of health status				
Very good	7.534(6.055–9.523)	422.341 (<.0001)	2.854(2.395–3.430)	170.730 (<.0001)
Good	6.800(5.492–8.559)	440.585 (<.0001)	2.061(1.743–2.457)	12.535 (0.0004)
Average	5.836(4.714–7.346)	229.016 (<.0001)	2.190(1.854–2.610)	40.467 (<.0001)
Poor	3.247(2.604–4.112)	34.758 (<.0001)	1.908(1.603–2.291)	0.0376 (0.8463)
Very poor	1		1	
EQ-5D-3 L				
Total	9.288(7.554–11.468)	437.979 (< 0.0001)	1.701(1.392–2.089)	26.312 (<.0001)
19–39	0.140(0.104–0.191)	157.9003 (< 0.0001)	0.024(0.017–0.033)	482.214 (<.0001)
40–64	0.899(0.638–1.286)	0.3574 (0.5500)	0.195(0.139–0.277)	86.317 (<.0001)
65 or older	7.086(2.611–21.201)	13.4291 (0.0002)	1.686(0.823–3.696)	1.8573 (0.1729)

^a^ Control group = no impairment group

^b^ Health-related quality of life

Regarding social activities, performing friendship activities once or more per month was significantly lower by 0.584-fold (95% CI = 0.566–0.602, *P* < 0.0001) in the Mild Impairment groups and 0.540-fold (95% CI = 0.520–0.561, *P* < 0.0001) in the Moderate-to-Severe Impairment groups compared to the No Impairment groups. Performing leisure activities once or more per month was significantly increased by 1.200-fold (95% CI = 1.164–1.238, *p* < 0.0001) in the Mild Impairment groups compared to the No Impairment groups (Table 3).

Regarding psychosocial factors, stress, depression, and suicidal ideation significantly increased the frequency of impairments in daily activities. In addition, the effect of suicide attempts was not significant in the Mild Impairment group, while Moderate-to-Severe Impairment group increased by 2.012-fold (95% CI = 1.549–2.571, *P* < 0.0001) (Table 3).

Among the age groups ranging from 19 to 64 years, the scores for the EQ-5D-3 L were significantly lower because the frequency of impairment increased (see Table 3). Conversely, among those aged 65 or older, the scores for the EQ-5D-3 L were 7.086-fold (95% CI = 2.611–21.201, *P* < 0.0002) higher in the Mild compared to the No Impairment group (Table 3).

## Discussion

In this study, we examined the frequency of impairments in daily activities and its associations with IGS overuse by age and impairment groups. Our results showed that for adults under the age of 60, the frequencies of daily activity impairments due to IGS overuse gradually decreased with increasing age. However, a higher proportion of adults aged 70 years or more were in the Moderate-to-Severe Impairment group due to IGS overuse than in the Mild Impairment group due to IGS overuse although these differences were not statistically significant. These findings should not be interpreted as implying that impairment of daily activities due to IGS overuse is currently severe among adults aged 70 years old or more. However, the frequency of impairments in daily activities due to IGS overuse was different among adults aged 70 or more and the other age groups, and we suggest the severity of impairments in daily activities due to IGS overuse in older adults will change in the near future. While usage rates of the Internet and social media among adults aged over 65 years have been rapidly increasing [58], research on the topic for this age group has lagged compared to research with younger populations [59]. Additionally, existing research has focused exclusively on the whether or not adults used the Internet or social media, not on their overdependence or addiction [59–61], while the physical and psychological effects of IGS overuse have been insufficiently investigated. Considering that smartphones were introduced in the 2000s [62], and that the early adults at that time will be middle- or old-aged in the next 10 years, we believe that more research should be conducted to investigate both the positive and/or negative effects of IGS overuse on various physical, psychological, and social factors among middle-aged and old-aged adults.

In this study, male participants showed a higher frequency of impairments in daily activities due to IGS overuse than female participants. Considering that such impairments are one of the criteria for IGS addiction [13, 18, 19], our results are highly indicative that men may be more inclined toward IGS addiction compared to women. Gender differences related to Internet or smartphone addiction are corroborated by previous research: The male gender has been associated with Internet addiction [24, 63], and longer time per week spent on the Internet for leisure purposes [7], such as online gaming [34, 35]. Generally, previous literature shows that women are more addicted to smartphones compared to men [22, 63], but there are some studies, which show that men are more addicted to smartphones compared to women [21, 64]. Aljomaa et al. reported that males tend to be more preoccupied with their smartphones, and were more likely to be negatively affected by them [21]. Currently, Internet overuse may be common among smartphone users [34], and Internet addiction is positively associated with smartphone addiction [63]. In previous studies, Internet-addicted groups tended to use the Internet via their mobile phones [35], and the preferred application among those addicted to the Internet was smartphone games, which is one of the potential factors related to smartphone addiction [65]. The use of gaming applications is higher for men than women [66]. Chen et al. found that playing smartphone games was a predictor of smartphone addiction for males [65], suggesting that men are more likely to be excessive users of IGS. This study also showed that males are likely to experience impairments in daily activities due to IGS overuse, which is one of the criteria for IGS addiction. Further research is needed to investigate how the use of the Internet, gaming, and smartphones are associated with one another, and whether males who are addicted to Internet and gaming are also more addicted to smartphones. One of the limitations in this study is that there was a higher proportion of 19–29 age group who were more likely to be addicted to online games [65, 66] than the other age groups, and we did not examine differences in the usage of smartphone applications by age groups.

Regarding alcohol and smoking, which are considered as substance abuse related to addictive behavior [12], our results showed that having a history of alcohol consumption in the year prior to the survey and being a current smoker were both associated with a higher frequency of impairments in daily activities due to IGS overuse. Sung et al. reported that increased alcohol use was associated with higher levels of smartphone addiction because both smartphone and alcohol usage facilitated social relationships [63]. Additionally, the Internet gaming disorder is a behavioral addiction that appears to be similar to substance-related addictions such as alcohol consumption [67], and Kim et al. found that the risk of internet addiction had a high association with smoking and alcohol consumption [24]. Conversely, some studies reported no association between IGS overuse and alcohol use or smoking [8, 13]. Therefore, these reported inconsistencies in past literature suggest that future studies should explore the gap in knowledge on the association between IGS overuse and substance use.

In the present study, the frequency of impairments in daily activities were the most severe among students, that is, generally among younger adults; this finding is corroborated by previous literature [15, 68]. However, regarding the associations between other sociodemographic variables (e.g. education and income) and Internet and smartphone addiction, our results showed some inconsistency when compared with those of previous literature [21, 69], probably owing to differences in cultural contexts between studies [66].

In the previous studies, IGS addiction has been found to negatively affect interpersonal relationships and social activities [22, 28, 70]. Contrastingly, our results showed that those with higher frequency of impairments in daily activities due to IGS overuse had a greater contact with friends—this result could be because the survey used for data collection considered that an individual could have had contact with friends both through calling and through personal meetings. Indeed, a previous study showed that one of the main reasons for using a smartphone is to access SNS and to utilize Internet-based instant messaging or chatting [6], which may strengthen the bonds between close friends as well as help people maintain their social connections [6, 15]. However, such activities reduce the value of spending face-to-face time with friends, leading to a decrease in the number of face-to-face meetings and social activities that require physical presence [15, 22, 70]. Similarly, our results showed that the number of gatherings aimed at solidifying friendships was quite low. Hence, we believe that one of the practical implications of this discussion may be that healthcare professionals in clinical or community settings serving those with IGS addiction consider providing information to their patients about comfortable places where they can meet friends, so that they do not forget the value of in-person social interactions and spending time with friends during their free time.

Similarly, the Mild and Moderate-to-Severe Impairment groups, which experienced a higher frequency of impairments in daily activities due to IGS overuse, spent more time on leisure activities than did the No Impairment group. This finding could reflect the trend of perceiving online and offline gaming and Internet activities through smartphones as being leisure activities [5, 9, 49]. This means that, in clinic or community settings, interventions aimed at adults who are addicted to IGS or who are facing severe impairments to their daily activities due to IGS overuse should consider limiting the length of time that certain mobile applications are allowed to be used for such leisure activities; this strategy was supported by previous research [71], and we further endorse it based on our results.

Psychosocial factors (e.g. stress, depression, suicidal ideation, and suicide attempt) showed statistically significant positive relationships with impairment in daily activities due to IGS overuse; several previous studies showed similar findings [6, 15, 27, 30, 32, 68, 70, 72, 73]. In this study, the Moderate-to-Severe Impairments group had higher levels of suicidal ideation and a higher number of suicide attempts compared to the two other groups. However, studies on addictive behavior and suicidal tendencies have concentrated primarily on Internet and gaming addiction [68, 74], and there is insufficient research on smartphone addiction: currently, research on the topic has focused on psychosocial discomfort [6, 21, 27], physical discomfort [25, 26], and the development of measurement instruments from the perspective of “behavioral addiction,” including compulsive behavior, maladaptive use, and functional impairment owing to smartphone overuse [4, 75]. Given that smartphones are becoming the primary medium for accessing the Internet and gaming [10], we suggest that future research examine the differences in psychological and physical variables (including suicidal tendencies) among individuals addicted to smartphones in order to help discern whether the addiction refers to the smartphone as a medium to the Internet and its resources, or to the smartphone itself.

Our results showed a general decrease in HRQoL with increasing age, and this finding has corroboration in previous literature [37]. Also consistent with previous research [30–32], our results showed that the higher the frequency of impairments in daily activities due to IGS overuse, the lower the HRQoL among Korean adults aged between 19 and 64. Moreover, our results showed that, among those aged over 65, the Mild Impairments group showed higher HRQoL in comparison to the No and Moderate-to-Severe Impairment groups. This finding could be considered when developing interventions/programs for older adults with low quality of life in clinical or community settings, in that Internet usage, light gaming, or ICT devices (e.g. smartphones) could be applied to enhance their quality of life. According to previous findings on the use of ICTs, older adults have been reported to decrease their negative emotions (e.g. loneliness) by increasing their connections to family and friends [59], and it has also been shown to reinforce older adults’ social activity via increased access to community resources [60], consequently improving physical and psychological well-being and life satisfaction. This means that older adult’s quality of life appears to be influenced by factors such as loneliness and lack of social participation, not merely by aging [76]. Additionally, from a physiological perspective, the use of ICTs among older adults may enhance the level of activities of daily living by allowing them to acquire new information and skills [59] and by reinforcing their ability to manage health problems and compensate for functional disabilities [61].

Although we do believe our study contributes to the current state of research on IGS addiction, our study still has a few limitations that we need to acknowledge: First, it was designed as a cross-sectional study, making it difficult to identify any causal relationships between the various studied variables and the frequency of impairments in daily activities due to IGS overuse. Second, the study was limited to a sample comprising Korean adults; thus, the findings cannot be generalized to other ethnic groups and should be applied to international contexts with care. Third, in the questionnaire used by the national survey from which we derived our data, the items assessing leisure activities did not distinguish between outdoor and indoor activities; hence, future research should make this distinction in the questionnaire to allow for the examination of any differences between indoor and outdoor activities and IGS overuse. Finally, the frequency of impairments in daily activities due to IGS overuse (one of the main variables in our study) was assessed using a single item, and we do acknowledge that merging internet, gaming, and smartphone usage into a single item within a questionnaire may cause many ambiguities to those responding to it. The Internet is a form of technology; however, a smartphone is an ICT device, and online games are software; therefore, our study does not allow for the examination as to which of these devices and/or technologies—and their usage—disturb the referenced daily activities.

However, this challenge with differentiation, at the same time, denotes one of the strengths of our study: To the best of our knowledge, this was the first study to investigate the relationship between the frequencies of impairment of daily activities due to IGS overuse and the physical, mental, and sociological factors of Korean adults through one single question. Second, we demonstrated the differences in HRQoL by IGS overuse and age-related differences. Third, the usefulness of the EQ-5D-3 L scale was confirmed when measuring HRQoL related to IGS overuse.

## Conclusions

This study evaluated the relationship between frequency of impairments in daily activities due to IGS overuse and physical, psychological, and sociological factors (including the HRQoL) of Korean adults using only one item: “In the past year, have your daily activities been disturbed because you were excessively using the Internet, gaming, or using a smartphone for leisure, and not work or learning purposes?” Our results showed that all age groups had their physical and psychological factors negatively influenced by IGS overuse. Further, among adults aged under 60 years, IGS overuse appeared to be negatively associated with higher HRQoL scores. Nonetheless, in the age group of 65 or older, higher frequencies of impairment in daily activities due to IGS overuse were positively associated with higher HRQoL scores. Future research should identify the causal relationships between various variables and IGS overuse among more age groups, including middle-aged and older adults, using the EQ-5D-3 L as a tool to assess HRQoL.

## Supplementary information

**Additional file 1: Supplementary File 1.** The analytic method of weight value.

**Additional file 2: Supplementary File 2.** Description of variables used in this study sociodemographic attributes, social relationships & activities, and psychosocial factors.

## Data Availability

The datasets analyzed during the current study are available from the corresponding author on reasonable request. Moreover, the raw datasets of the 2017 Community Health Survey analyzed during the current study are available in the Korea centers for Disease Control and Prevention website: https://chs.cdc.go.kr/chs/index.do.

## Abbreviations

ICT: Information and communication technology
IGS: Internet, gaming, and smartphone
HRQoL: Health-related quality of life
EQ-5D-3 L: EuroQol-five-dimensions three-level version
SNS: Social networking services
WHO: World Health Organization
ICD-11: the 11th revision of the International Classification of Diseases
DSM-5: the fifth edition of the Diagnostic and Statistical Manual of Mental Disorders
