# Supplementary material for: The frequency of impairments in everyday activities due to the overuse of the internet, gaming, or smartphone, and its relationship to health-related quality of life in Korea
Source: BMC Public Health. 2020 Jun 18;20:954. doi: 10.1186/s12889-020-08922-z (PMC7301989; doi:10.1186/s12889-020-08922-z)
Supplement: Supplementary file 1 — Additional file 1: Supplementary File 1. The analytic method of weight value. [file 12889_2020_8922_MOESM1_ESM.docx]

**Supplementary File 1.** [The analytic method of weight value]

**Article title:** The frequency of impairments in everyday activities due to the overuse of the internet, gaming, or smartphone, and its relationship to health-related quality of life in Korea

**Journal name:** BMC Public Health

**Author names:** Yeo-Won Jeong RN, PhD^*^, Young-Ran Han, RN, PhD, Sang-Kyu Kim MD, PhD, Han-Seok Jeong, master of course student

**Corresponding author**

Yeo-Won Jeong, RN, Ph.D.

Address: Department of Nursing, Dongguk University College of Medicine, 123 Dongdae-ro, Gyeongju-si, Gyeongsangbuk-do, 38066, Republic of Korea

Tel. 82-54-703-7805

Fax. 82-54-770-2616

E-mail: ywjeong@dongguk.ac.kr

Each weight value was calculated as below;

1. Household weight: Weight reflecting the ratio of household extraction, the ratio of eligible household, and the ratio of household by type of housing considering the process of sampling extraction of sample design

*
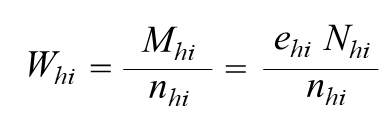
*

*M_hi_* = *i* number of eligible household by housing type (general house or Apartment) in *h* (district/street/village)

*n_hi_* = *i* number of surveyed household in *h* (district/street/village)

*e_hi_* = *i* eligible household ratio for survey in *h* (district/street/village)

*N_hi_* = *i* number of household by housing type (general house or Apartment) in *h* (district/street/village)

2. Personal weight: Weight reflecting the individual response rate to household weight

*
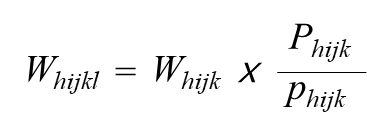
*

*h*: district/street/village

*i*: type of housing (General house or Apartment)

*j*: sample area

*k*; household

*l*; the number of household member

*P_hijk_*: the number of household members aged 19 or older

*p_hijk_*: the number of surveyed household members aged 19 or older

(3) Adjusted weight: Weight value being adjusted the population structure by sex and age group based on the resident registration population


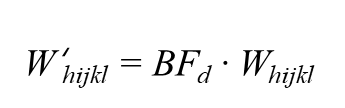


*
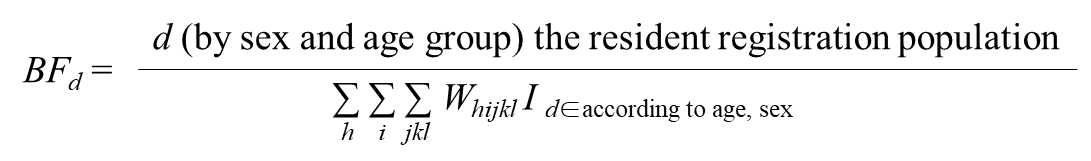
*
