# Supplementary material for: The frequency of impairments in everyday activities due to the overuse of the internet, gaming, or smartphone, and its relationship to health-related quality of life in Korea
Source: BMC Public Health. 2020 Jun 18;20:954. doi: 10.1186/s12889-020-08922-z (PMC7301989; doi:10.1186/s12889-020-08922-z)
Supplement: Supplementary file 2 — Additional file 2: Supplementary File 2. Description of variables used in this study sociodemographic attributes, social relationships & activities, and psychosocial factors. [file 12889_2020_8922_MOESM2_ESM.docx]

**Supplementary File 2.** [Description of variables used in this study _ sociodemographic attributes, social relationships & activities, and psychosocial factors]

**Article title:** The frequency of impairments in everyday activities due to the overuse of the internet, gaming, or smartphone, and its relationship to health-related quality of life in Korea

**Journal name:** BMC Public Health

**Author names:** Yeo-Won Jeong RN, PhD^*^, Young-Ran Han, RN, PhD, Sang-Kyu Kim MD, PhD, Han-Seok Jeong, master of course student

**Corresponding author**

Yeo-Won Jeong, RN, Ph.D.

Address: Department of Nursing, Dongguk University College of Medicine, 123 Dongdae-ro, Gyeongju-si, Gyeongsangbuk-do, 38066, Republic of Korea

Tel. 82-54-703-7805

Fax. 82-54-770-2616

E-mail: ywjeong@dongguk.ac.kr

The sociodemographic attributes examined in this study included gender, age, education level, smoking status, drinking history, occupation, marital status, average monthly income, and area of residence. Age was grouped initially into 19-39, 40-64, and 65 or older. Education level consisted of 4 categories: no formal education or elementary school education, middle school education, high school education, and college or higher (including university/graduate school education). Marital status consisted of 3 categories: [married and living with a spouse]; [divorced, widowed, or separated], and [never been married]. Average monthly household income consisted of 4 categories: lowest (under 1 million Korean won), low-middle (over 1 million but less than 3 million Korean won); upper-middle (over 3 million but less than 5 million Korean won), and highest (over 5 million Korean won). Occupation consisted of 6 categories according to physical activity level: [manager, professional, or administrator; clerk], [service worker; sales person], [technician, mechanic, or production worker; machine operator/assembly worker], [skilled agricultural/forestry/fishery worker; unskilled worker; soldier], [student], and [unemployed]. Smoking status consisted of 3 categories: non-smoker, past smoker, and current smoker. History of drinking consisted of 2 categories: no drinking (in the past year) and drinking (at least once in the past year). Area of residence was classified as rural if the respondent lived in a town/township and as urban if the respondent lived in a city neighborhood.

Items regarding social relationships & activities inquired about the frequency of contact with family/neighbors/friends and religious/friendship/leisure/charity activities. The frequency of contact with family/neighbors/friends were assessed with the three items, “How many times do you meet or call with 1) your family including relatives/2) neighbors/3) friends who you are a lot of contacting?” Contacts with family, neighbors, and friends by meeting in person or calling consisted of 4 categories: once or less per month, 2-4 times per month, 1-3 times per week, and 4 times or more per week. Religious, friendship (outdoor), leisure, and charity activities were assessed with the four items, “Do you attend 1) religious activity/2) friendship activity (outdoor activity, ex. Alumni meeting)/3) leisure activity/4) charity activity once a month regularly?” Responses to the four items were categorized into 2 groups: less than once per month and once or more per month. With the exception of friendship activities, the remaining 3 types were not specified as either indoor or outdoor activity.

Psychosocial factors included stress, depression, suicidal ideation, and suicide attempt. Stress was assessed with the item, “How stressed are you when performing an everyday activity?” Responses to this item were grouped into one of the following 4 categories: no stress, some stress, moderate stress, and severe stress. Depression, suicidal ideation, and suicide attempt were assessed with the items, “In the past year, have you felt sadness or despair for 2 consecutive weeks or longer, such that everyday activities were interrupted?”; “In the past year, has it occurred to you that you wanted to die?”; “In the past year, have you attempted to commit suicide?” respectively. The responses to each of the items were either yes or no.
